# Supplementary material for: Aspirin-responsive gene switch regulating therapeutic protein expression
Source: Nat Commun. 2025 Feb 27;16:2028. doi: 10.1038/s41467-025-57275-x (PMC11868571; doi:10.1038/s41467-025-57275-x)

# Supplementary Information

## Aspirin-responsive gene switch regulating therapeutic protein expression

Jinbo Huang[1], Ana Palma Teixeira[1], Ting Gao[2], Shuai Xue[1,2], Mingqi Xie[2,3], Martin Fussenegger[1,4,*]

[1]Department of Biosystems Science and Engineering, ETH Zurich, Klingelbergstrasse 48, CH-4056 Basel, Switzerland.

[2]Westlake Laboratory of Life Sciences and Biomedicine, Hangzhou, Zhejiang, China.

[3]Key Laboratory of Growth Regulation and Translational Research of Zhejiang Province, School of Medicine and School of Life Sciences, Westlake University, Hangzhou, Zhejiang, China.

[4]Faculty of Science, University of Basel, Klingelbergstrasse 48, CH-4056 Basel, Switzerland.
[*]Corresponding author. E-mail: fussenegger@bsse.ethz.ch

This file includes:

- Supplementary Figures: 1-17
- Supplementary Table: 1
- References
- Source data files for western blot and CO-IP

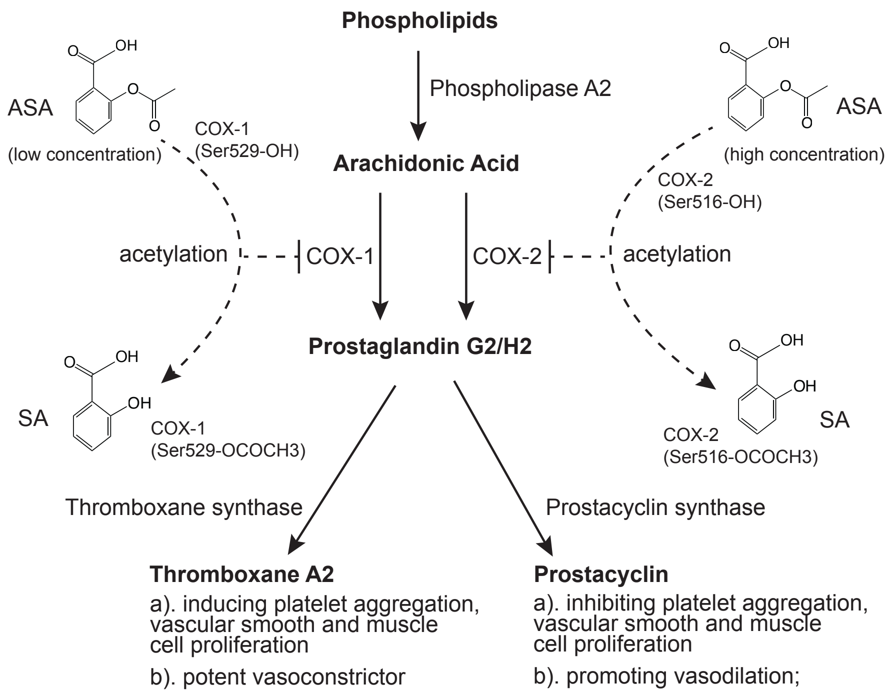


**Supplementary Figure 1. Mechanisms of action of acetylsalicylic acid (ASA, aspirin) on COX enzymes in humans.** Phospholipase A2 catalyzes the hydrolysis of membrane phospholipids to release arachidonic acid, a substrate for prostaglandin synthesis. Cyclooxygenase enzymes (COX-1 and COX-2) convert arachidonic acid into prostaglandin G2/H2, the precursors for bioactive prostanoids, including thromboxane A2 (via thromboxane synthase) and prostacyclin (via prostacyclin synthase) (solid arrows). Aspirin irreversibly inhibits COX-1 and COX-2 by acetylating Ser-529 and Ser-516, respectively, thereby blocking prostaglandin production (dashed arrows) [1-3].

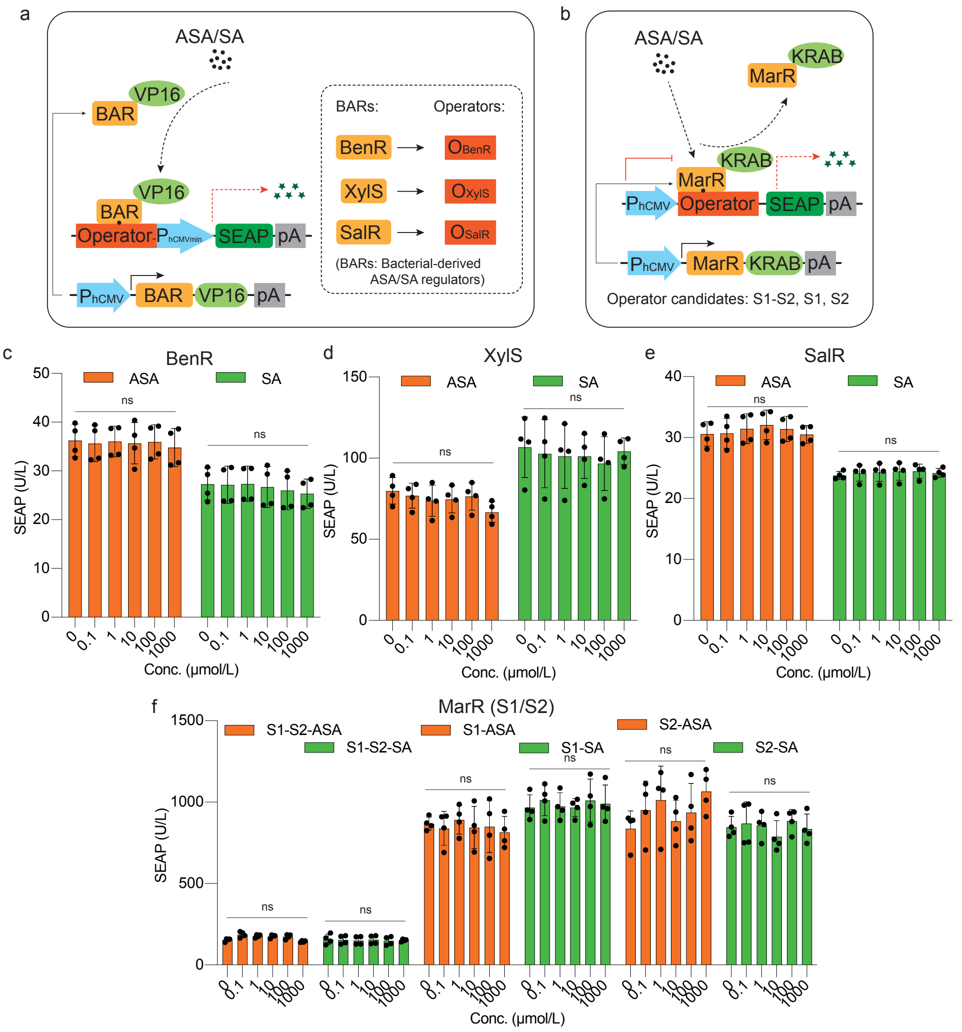


**Supplementary Figure 2 | Exploring the potential of various bacterial-derived ASA/SA sensors as components of mammalian gene switches inducible by aspirin. a-b**, Schematic depiction of the constructs used to test the potential of bacterial-derived ASA/SA regulators (BARs) for constructing aspirin-inducible gene switches in mammalian cells. BARs utilized in this study include SalR (salicylate-dependent transcriptional regulator), BenR (benzoate response regulator), XylS (xylene degradation regulatory protein S), and MarR (multiple antibiotic resistance regulator). In **a**, each BAR was fused to the transactivation domain VP16 from the herpes simplex virus early transcriptional activator. In the absence of aspirin (ASA) or SA, the constitutively expressed BAR-VP16 fusion protein cannot bind to its cognate DNA operator, resulting in the suppression of reporter gene (SEAP) expression (solid arrows). Conversely, the presence of the inducers enables BAR to engage with its cognate DNA operators, facilitating subsequent activation of reporter gene (SEAP) expression (dashed arrows). In **b**, MarR (multiple antibiotic resistance regulator) was fused with a trans-silencer domain (KRAB) that can bind to the $O_{MarR}$ (S1-S2, S1, S2) sequence placed downstream of a constitutive promoter ($P_{hCMV}$). In the absence of ASA or SA, transgene expression is prevented

(solid lines). In the presence of ASA or SA, the synthetic repressor loses affinity for $O_{MarR}$ and the reporter expression is switched on (dashed arrows). **c-f**, Evaluation of the gene switches based on BenR (**c**), XylS (**d**), SalR (**e**) and MarR (**f**) upon induction with ASA and SA at the indicated concentrations. Levels of secreted alkaline phosphatase (SEAP) in the culture supernatants were measured 24 hours post-induction. Statistical significance was analysed by means of a two-sided unpaired t test. Data points represent mean ± SD; n = 4. "ns" denotes not significant (*P* value > 0.05). The *P* value indicates the significance of differences in mean values versus the uninduced group. Source data are provided as a Source Data file.

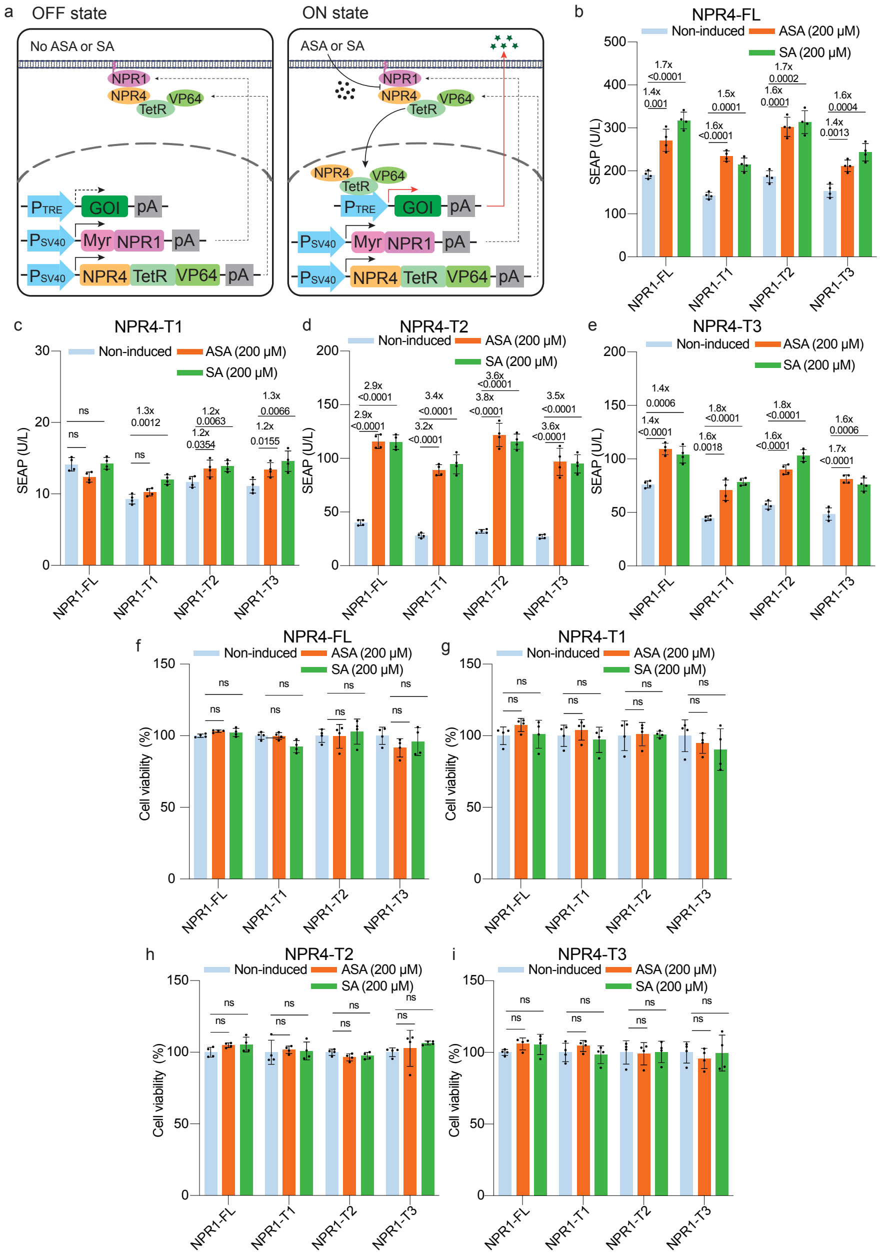


**Supplementary Figure 3 | Design and optimization of the ASA/SA-induced system in engineered HEK-293T cells. a,** Schematic representation of the genetic components required

for constructing an ASA-inducible gene switch in mammalian cells. In the absence of ASA or SA, constitutively expressed NPR1 containing a plasma-membrane-anchored myristoylation signal peptide (Myr) is anchored to the cell membrane, where it interacts with constitutively expressed NPR4 fused to the tetracycline-dependent transrepressor TetR and the transactivation domain VP64 (four tandem repeats derived from VP16), resulting in the silencing of reporter (SEAP) expression under the TRE (TetR responsive element). Conversely, in the presence of the inducer (ON state), binding disrupts the NPR1/NPR4 complex interaction, releasing NPR4-TetR-VP64 from the cell membrane and enabling its translocation to the nucleus. There it binds to a synthetic promoter containing TetR-responsive elements (TRE), activating expression of the reporter gene (SEAP). **b-e**, Screening of NPR1 and NPR4 truncation variants (see **Figure 1b**). HEK-293T cells co-transfected with the indicated NPR1 and NPR4 truncation variants were treated with or without ASA/SA (200 μM) and SEAP levels in the culture supernatants were measured 24 h after treatment. **f-i**, Impact of ASA or SA on the viability of HEK-293T cells co-transfected with NPR1 and NPR4 truncation variants. Cells were exposed to ASA and SA at the indicated concentrations, and cell viability was measured using resazurin assay. Statistical significance was analysed by means of a two-sided unpaired t test. Data points represent mean ± SD; n = 4. ns means not significant (*P* value > 0.05). The *P* value indicates the significance of differences in the mean values versus the indicated group. Source data are provided as a Source Data file.

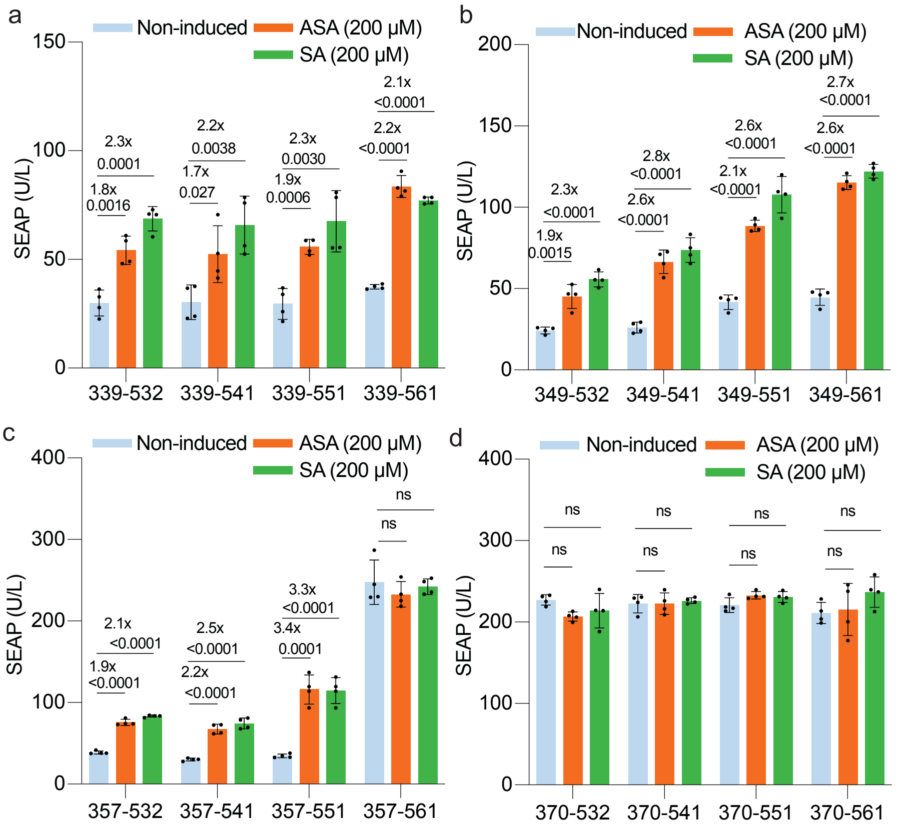


**Supplementary Figure 4 | Screening of NPR1 and NPR4 truncation variants.** HEK-293T cells were co-transfected with the indicated NPR1 and NPR4 truncation variants based on the results in **Supplementary Figure 2d**. SEAP levels in the culture supernatants were measured at 24 h after induction. Cells were exposed to ASA and SA at the indicated concentrations. Data points represent mean ± SD; n = 4. ns means not significant (*P* value > 0.05). Statistical significance was analysed by means of a two-sided unpaired t test. The *P* value indicates the significance of differences in the mean values versus the indicated group. Source data are provided as a Source Data file.

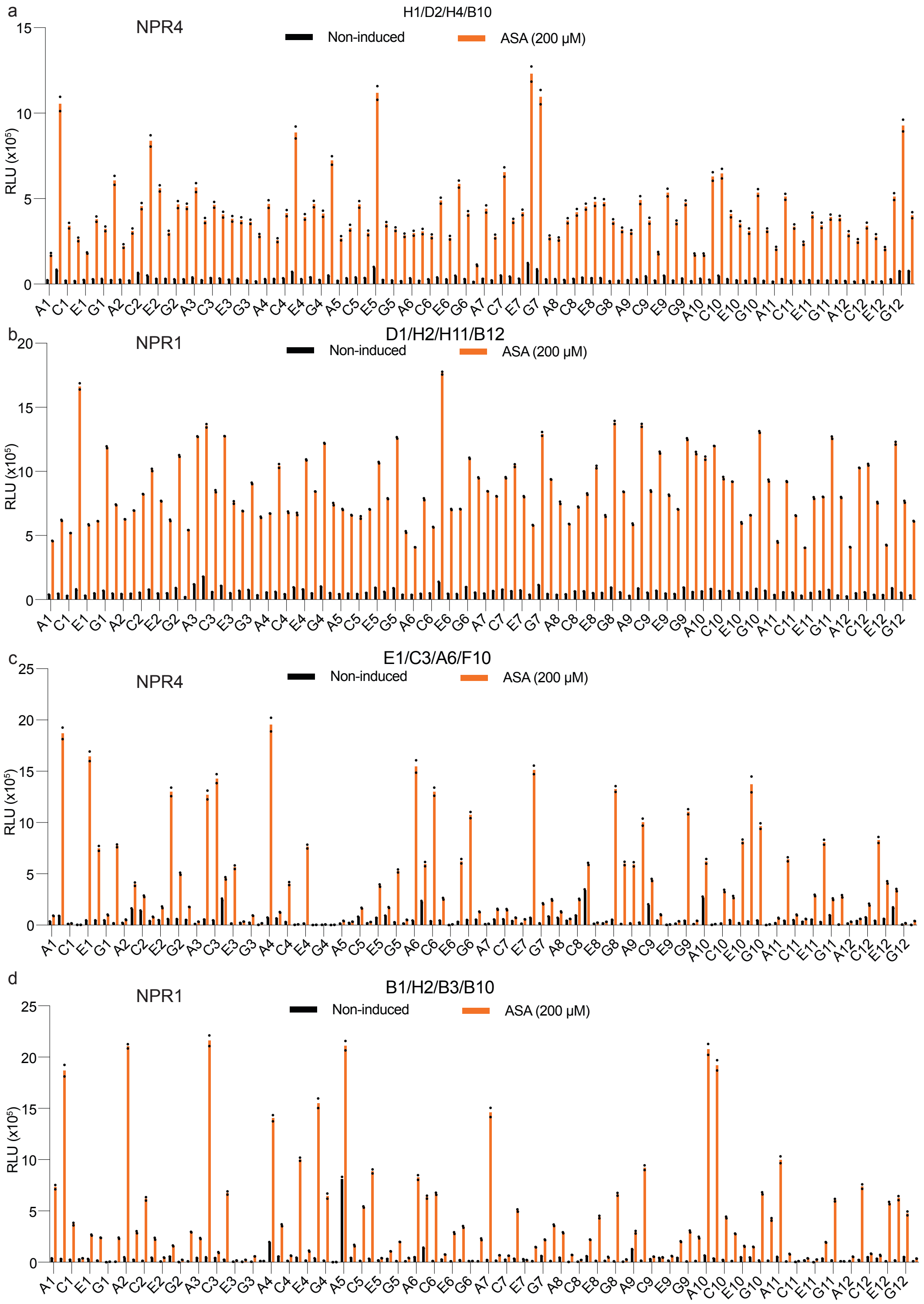


**Supplementary Figure 5 | Primary screening for mutagenesis and high-throughput assessment of mutant variants. a-d**, Primary screening of Myr-NPR1-T2 and NPR4-T2-VanR-VP16 plasmid libraries. NLuc levels were analyzed in non-induced (black) and induced (200 µM ASA, red) groups. The variants with good performance in terms of fold changes were selected for next round evaluation. Source data are provided as a Source Data file.

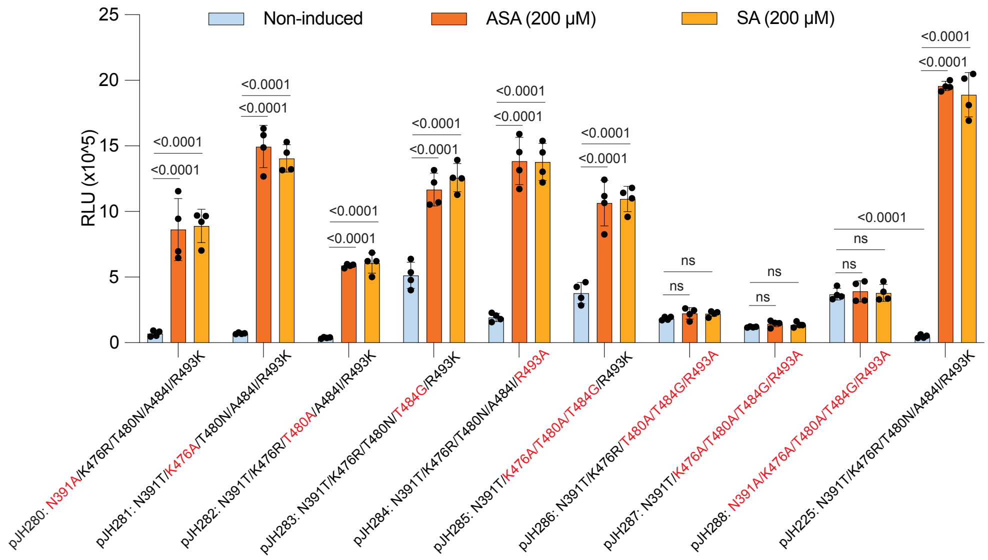


**Supplementary Figure 6. Reporter expression profiling of NPR4 bearing point mutations to alanine or glycine.** Cells were transfected with the indicated NPR4 mutant plasmids, NPR1$_{mut6}$, and the reporter plasmid pJH2012. The last construct, NPR4-T2 (NPR4$_{mut6}$), which exhibited the best performance from the directed evolution screening, contains five mutations: N391T/K476R/T480N/A484I/R493K. The mutated residues in the other transfected cells are shown in red. Statistical significance was analysed by means of two-sided two-way ANOVA with Tukey's test between groups and two-sided unpaired t test within groups. Data are presented as means ± SD (n = 4). "ns" means not significant ($P$ value > 0.05). $P$-values were calculated relative to the corresponding non-induced control. Source data are provided as a Source Data file.

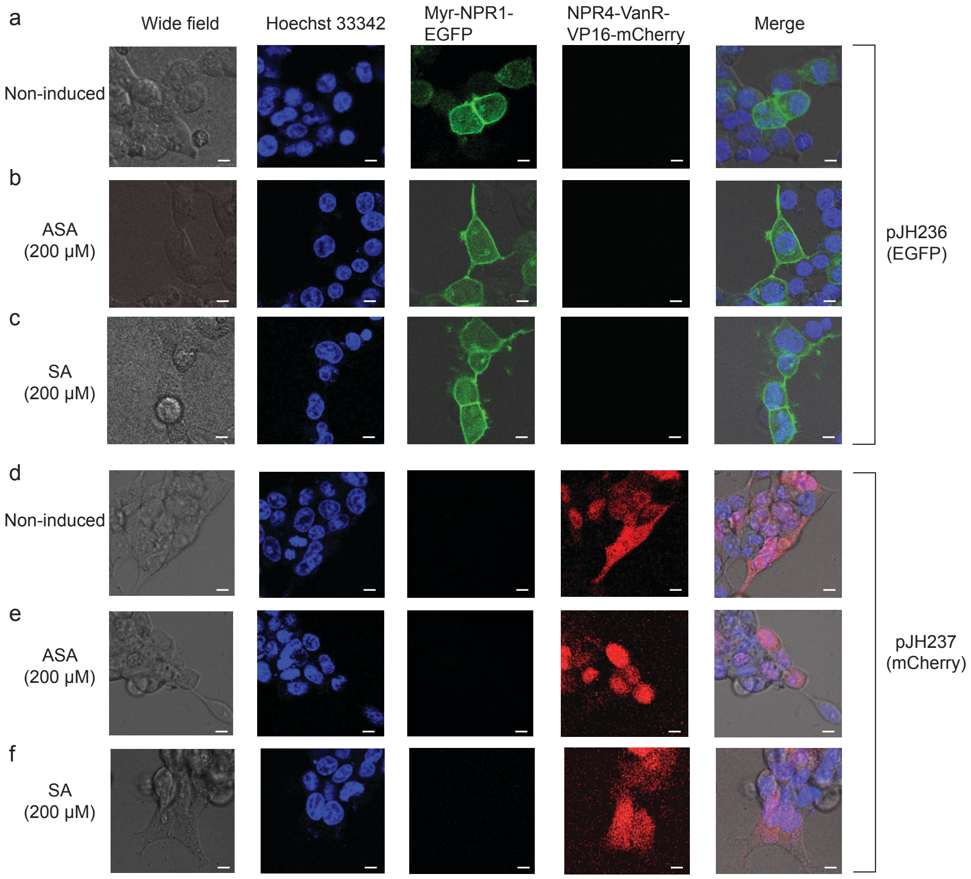


**Supplementary Figure 7 | Microscopy analysis of the ASPIRIN components. a-d,** HEK-293T cells were co-transfected with pJH2012 ($O_{VanO2}$-$P_{hCMVmin}$-NLuc-pA) and either pJH236 ($P_{SV40}$-Myr-NPR1-T2-mut6-EGFP-pA) (**a-c**) or pJH237 ($P_{SV40}$-NPR4-T2-mut6-VanR-VP16-mCherry-pA) (**d-f**). The engineered cells were treated with ASA (**b,e**) or SA (**c,f**). Control groups (**a,d**) were incubated with an equivalent amount of vehicle (50% DMSO). Cell nuclei were stained with Hoechst 33342. A representative image of three replicates from each group is shown. Scale bar (white): 10 μm.

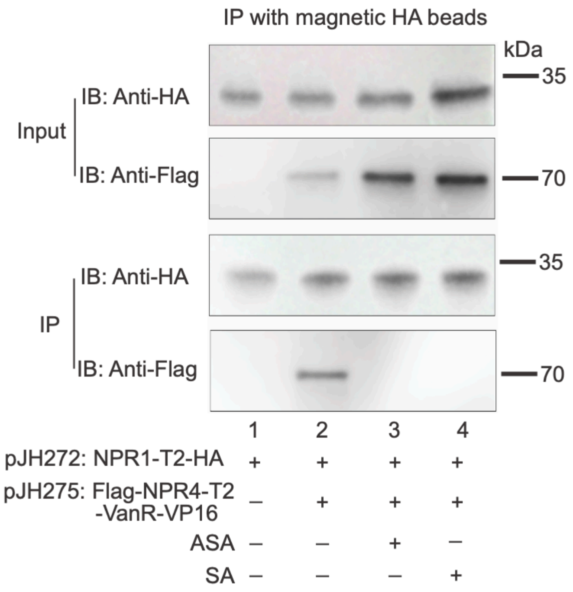


**Supplementary Figure 8. Detection of protein interactions between NPR1 and NPR4 using co-immunoprecipitation and Western blotting.** The interaction between NPR1-T2-HA (pJH272) and FLAG-NPR4-T2-VanR-VP16 (pJH275) was analyzed by co-immunoprecipitation (Co-IP) and Western blotting. HEK-293T cells were co-transfected with the two plasmids for 24 h, either in the presence or absence of ASA or SA. For treatment groups, cells were further incubated with fresh DMEM containing 200 µmol/L ASA or SA for 1 hour prior to cell lysis. Then the cells were lysed with cell lysis buffer, and the lysates were centrifuged to remove debris. The input samples were collected from the supernatant. Co-IP was performed using the Pierce™ HA-Tag Magnetic IP/Co-IP Kit (Thermo Fisher, cat. no. 88838) according to the manufacturer's protocol. Immunoprecipitated samples were subjected to Western blotting using antibodies against HA and FLAG epitopes. The concentration of ASA or SA is 200 µmol/L. Protein markers are indicated alongside the blot images. Abbreviations: IP, immunoprecipitation; IB, immunoblotting; HA, hemagglutinin tag; FLAG, FLAG octapeptide tag. Source data are provided in the end of this document.

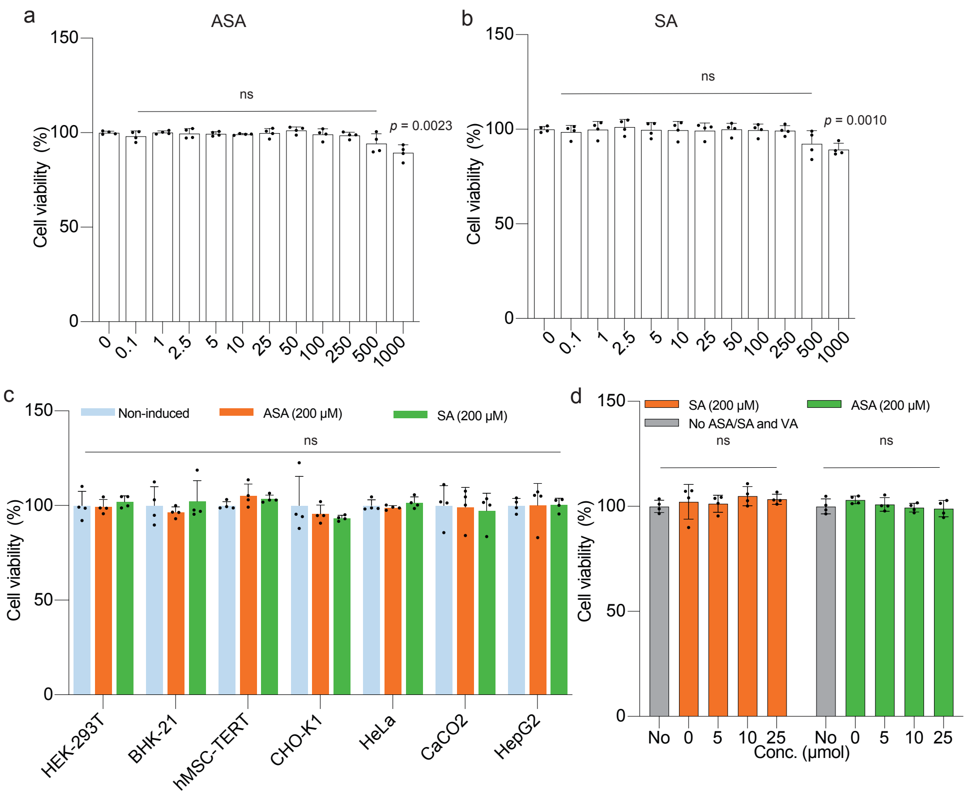


**Supplementary Figure 9 | Effects of ASA and SA on the viability of mammalian cells.** Cell viability was measured using the resazurin assay. All treatment groups were incubated with 200 μM ASA or SA, and the non-treated groups received an equivalent amount of vehicle (in 50% DMSO). In **d**, "No" means no inducer or vanillic acid was added in these groups. Data points represent mean ± SD; n = 4. ns means not significant ($P > 0.05$). Statistical significance was analysed by means of a two-sided unpaired t test. The $P$ value indicates the significance of differences in the mean values versus the non-induced group. Source data are provided as a Source Data file.

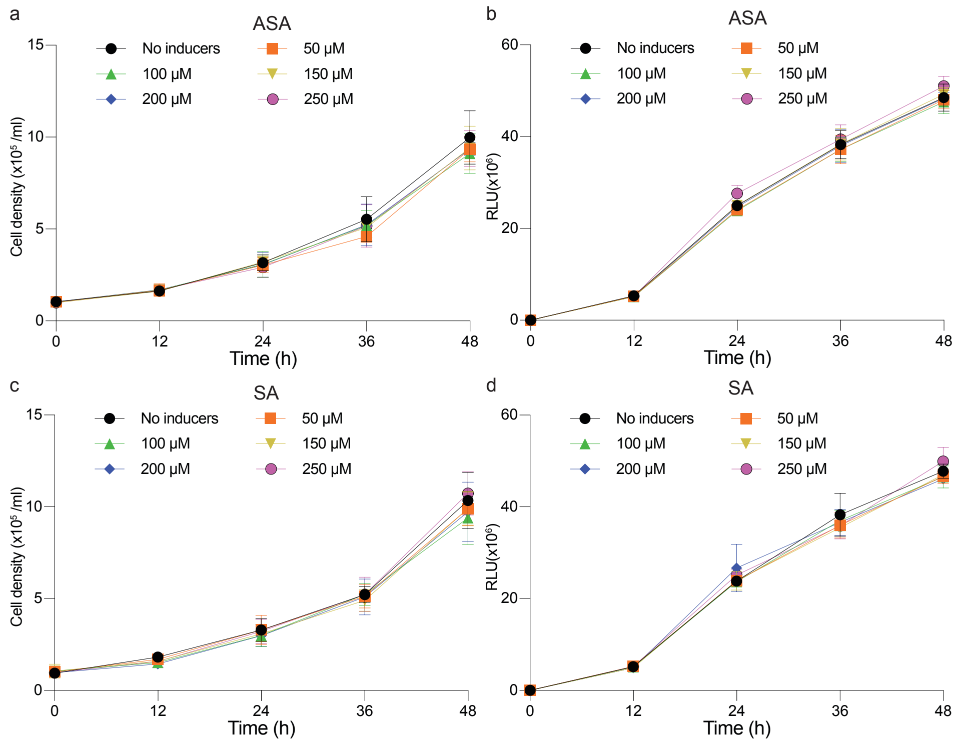


**Supplementary Figure 10 | Assessment of cell growth and recombinant protein production in the presence of ASA or SA.** (**a,c**) HEK-293 cells were seeded 24 h prior to incubation with the indicated concentrations of ASA (a) and SA (c). (**b,d**) Two million HEK-293T cells were seeded in a 10 cm dish overnight before transfection. Subsequently, the cells were transfected with 30 ng of pJH57, constitutively expressing NLuc in (**b**) and (**d**). Following 12 h of transfection, cells were evenly distributed into two 96-well plates for 12 h. Then, the cell culture medium was replaced with specified DMEM medium containing the indicated inducers or vehicle. Cell density (**a,c**) and recombinant NLuc production (**b,d**) were analyzed every 12 h during 48 h of culture. No significant difference observed in the treatment groups compared to the non-induced group ($P > 0.05$). Statistical significance was analysed by means of a two-sided unpaired t test. Data points represent mean ± SD, n = 4. Source data are provided as a Source Data file.

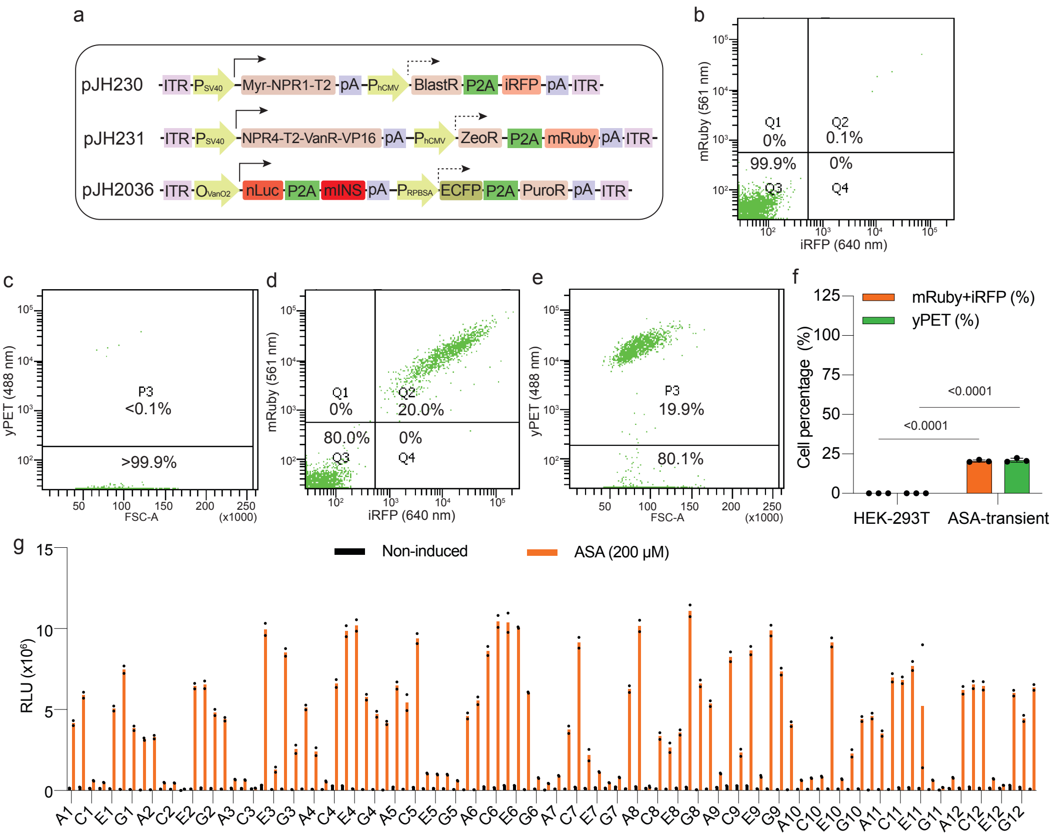


**Supplementary Figure 11. Generation and characterization of monoclonal cell line. a,** Design of plasmids used for the generation of monoclonal cell lines. The stable cell line constitutively expresses engineered Myr-NPR1-T2 (ITR-$P_{SV40}$-Myr-NPR1-T2-mut6-pA:$P_{hCMV}$-BlastR-P2A-iRFP-pA-ITR, pJH230), NPR4-T2-VanR-VP16 (ITR-$P_{SV40}$-AtNPR4-T2-mut6-VanR-VP16-pA: $P_{hCMV}$-ZeoR-P2A-mRuby-pA-ITR, pJH231) and tandem VanR operator-controlled NLuc followed by mouse insulin (mINS) (ITR-$O_{VanO2}$-$P_{hCMVmin}$-NLuc-P2A-mINS-pA: $P_{RPBSA}$-ECFP-P2A-PuroR-pA-ITR, pJH2036). All the constructs contain flanking inverted terminal repeats (ITR) for the recognition of SB100X transposase. **b-f**, Gating strategies of fluorescence-activated cell sorting (FACS) analysis for monoclonal cell lines using mRuby (558/605), iRFP (690/713) and yPET (517/530). **b-c**, human HEK-293T cells without transfection were used as a negative control. **d-e**, Human HEK-293T cells cotransfected with pJH230, pJH231, pJH2036 and SB100X transposase were sorted for selection of genomic integration of the ASA-induced system using mRuby (558/605), iRFP (690/713) and yPET (517/530). Triple-positive monoclonal cells were selected for two-week culture in medium containing 0.5 μg/ml puromycin, 5 μg/ml blasticidin and 100 μg/ml of zeocin for antibiotic selection. **f**, Bar plotting for Q2 (double positive for mRuby and iRFP, orange) and P3 (yPET positive, green) gates among negative control and targeting samples. **g**, Screening of monoclonal HEK-293T cell lines. HEK-293T cells were stably transfected with pJH230,

pJH231, pJH2036 and SB100X transposase. NLuc production was profiled from two 96-well plates of monoclonal cell lines after induction with ASA (200 µM) for 24 h. 10,000 cells were analyzed in **b-f**. In **f**, Data points represent mean ± SD; n = 3, and statistical significance was analysed by means of a two-sided unpaired t test. The *P* value indicates the significance of differences in the mean values versus the indicated group. Source data are provided as a Source Data file.

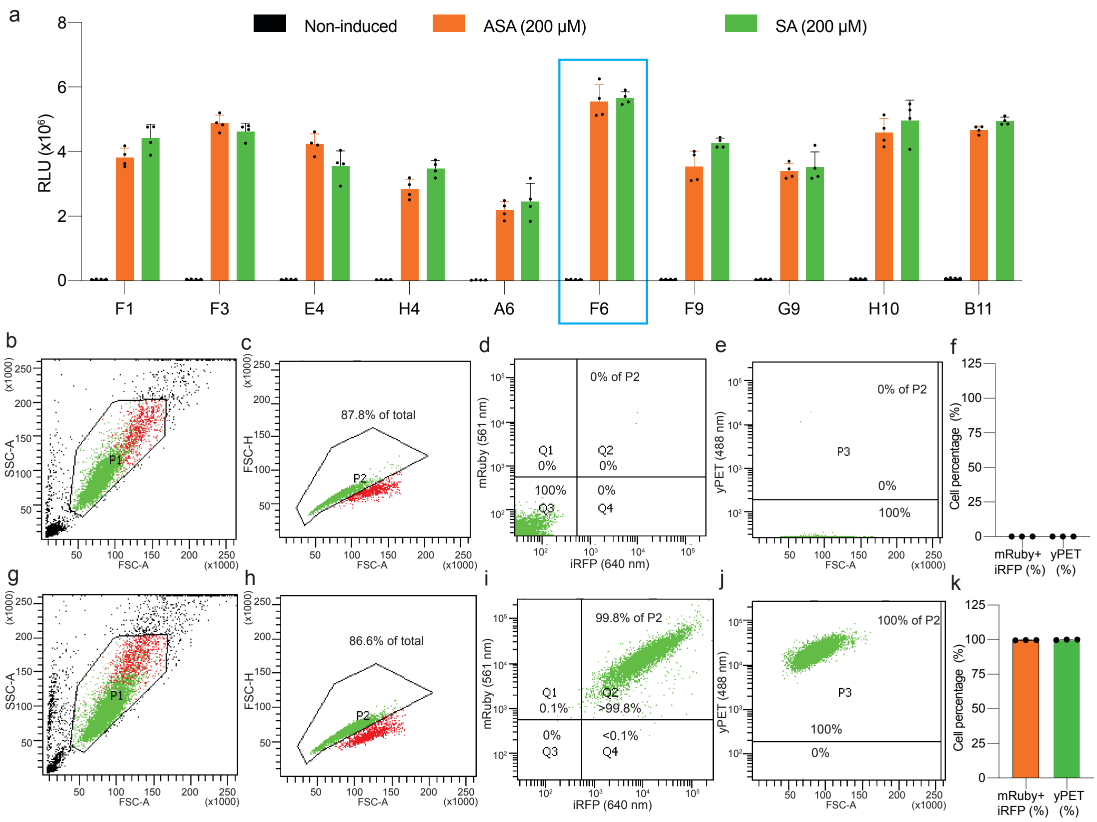


**Supplementary Figure 12. Secondary screening and confirmation of monoclonal cell line. a,** Evaluation of the top clones obtained from the plate in **Supplementary Fig. 8** involved quantification of NLuc levels in the culture supernatant after a 24-hour induction period. The most promising monoclonal cell line, F6, designated HEK-ASPIRIN and highlighted in blue, was selected for further experiments. **b-k**, Flow cytometric analysis of HEK-ASPIRIN monoclonal cells was conducted using mRuby (558/605), iRFP (690/713), and yPET (517/530). **b-e**, Gating strategies were implemented for human HEK-293T cells without transfection, serving as a negative control. **f**, Bar plotting for Q2 (double positive for mRuby and iRFP) and P3 (yPET positive) gates in the negative control demonstrated negligible fluorescence. **g-j**, Fluorescence analysis of the cell population using the same markers revealed a triple-positive population of 99.8%, confirming that HEK-ASPIRIN is indeed a monoclonal cell line. **k**, Bar

plotting for Q2 (double positive for mRuby and iRFP, orange) and P3 (yPET positive, green) gates among targeting cells indicated a high degree of fluorescence positivity. All data are presented as means ± SD; in **a**, n = 4; in **f** and **k**, n = 3. For all groups in **a**, all induction groups differ significantly from the corresponding non-induced groups ($P < 0.001$). Statistical significance was analysed by means of a two-sided unpaired t test. The $P$ values were calculated for the differences between the control and induced groups. 10,000 cells were analysed in **b-f** and **g-k**, respectively. Source data are provided as a Source Data file.

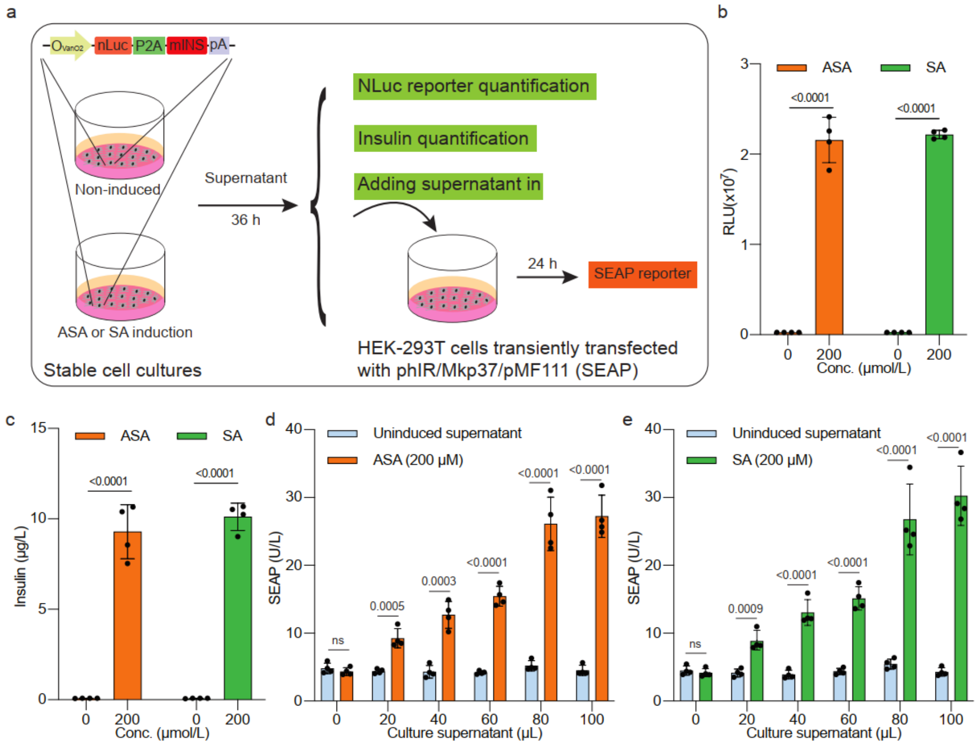


**Supplementary Figure 13 | Scheme and characterization of insulin-activity bioassay. a**, Schematic representation of the insulin activity bioassay. HEK-ASPIRIN stable cells were cultured in medium containing inducer or without inducer for 36 hours. NLuc and insulin quantification was performed, and the supernatant was subsequently transferred to HEK-293T cells transiently transfected with phIR ($P_{hCMV}$-hIR-pA), Mkp37 ($P_{hCMV}$-TetR-Elk1-pA) and pMF111 ($P_{TRE}$-SEAP-pA), then SEAP reporter in the culture supernatant was quantified after 24 h. **b**,**c**, Levels of NLuc (**b**) and insulin (**c**) in the culture supernatant of HEK-ASPIRIN stable cells after 36 h of induction. **d**,**e**, SEAP levels in insulin-activity bioassay as a readout of insulin activity after addition of the supernatant from the stable cell culture induced by ASA (**d**) and SA (**e**). SEAP in the culture supernatant was quantified after the addition of cell culture supernatant for 24 hours. RLU: relative luminescence units. Abbreviations for constructs are

listed after Supplementary Data 1. Statistical significance was analysed by means of two-sided two-way ANOVA with Tukey's test. Data points represent mean ± SD; n = 4. "ns" means not significant ($P > 0.05$). The $P$ value indicates the significance of differences in the mean values (versus the indicated group). Source data are provided as a Source Data file.

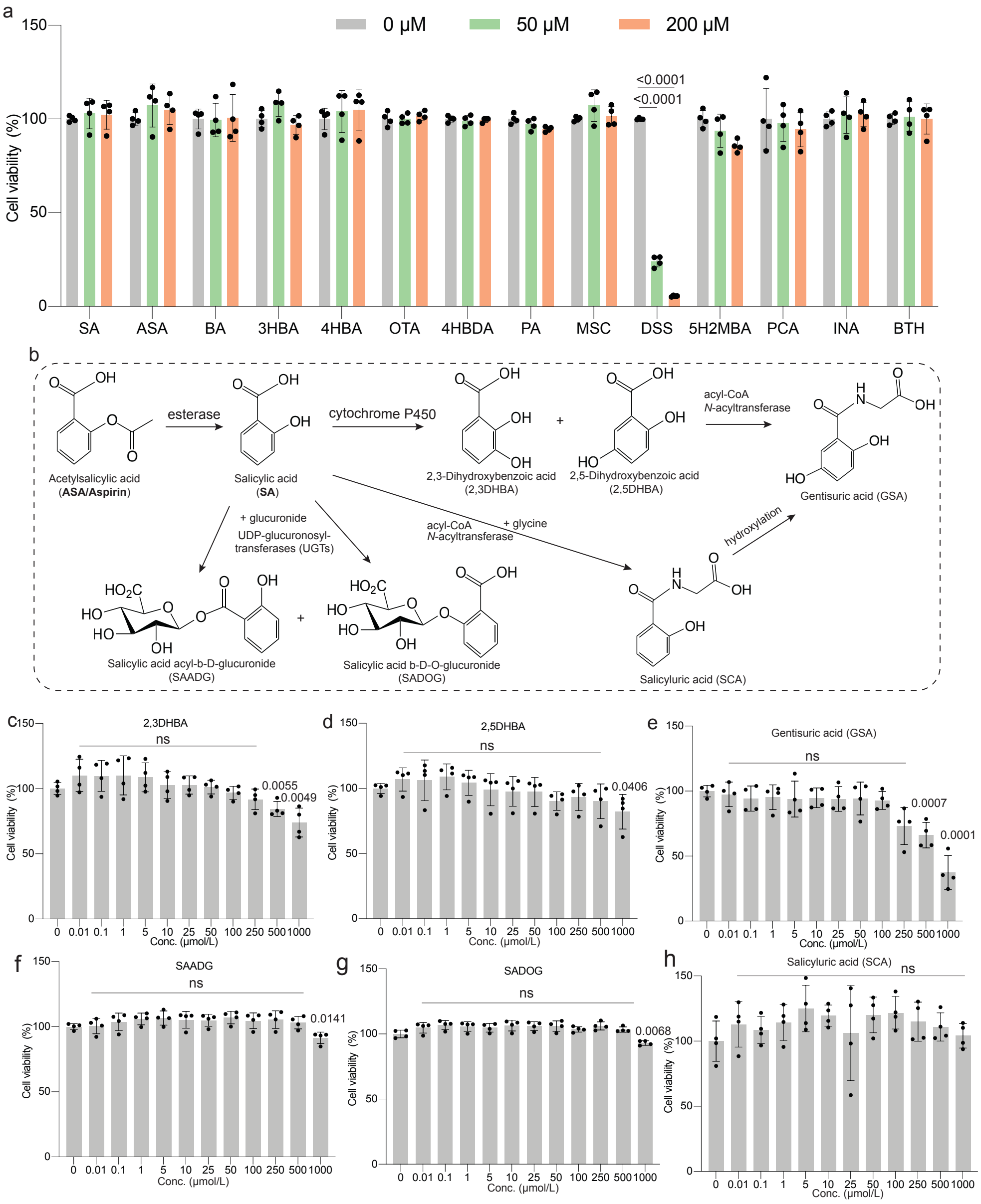


**Supplementary Figure 14 | Effects of ASA and SA on the viability of cells and ASA metabolism in mammals.** Viability was measured using resazurin assay in (**a**) and (**c-h**). **b**, ASA metabolism in mammals. ASA is hydrolyzed to SA by esterase. Subsequently, SA can follow various metabolic pathways, yielding diverse metabolites. Major products include salicyluric acid and glucuronides, catalyzed by acyl-CoA *N*-acyltransferase and UDP-glucuronosyltransferase (UGTs), respectively. SA can also undergo oxidation by cytochrome P450, producing 2,3-dihydroxybenzoic acid (2,3DHBA) and 2,5-dihydroxybenzoic acid

(2,5DHBA). In addition, acyl-CoA *N*-acyltransferase acts on 2,5-dihydroxybenzoic acid (gentisic acid) to afford gentisuric acid, which is also a hydroxylation product of SA[4,5]. 2,3DHBA, 2,3-dihydroxybenzoic acid; 2,5DHBA, 2,5-dihydroxybenzoic acid (Gentisic acid); SAADG, SA acyl-b-D-glucuronide; SADOG, SA b-D-O-glucuronide. Statistical significance was analysed by means of a two-sided unpaired t test. All data are means ± SD; n = 4. In **a**, apart from DSS group, none of the other chemicals showed a significant impact on cell viability compared to the non-induced controls ($P > 0.05$). "ns" means not significant ($P > 0.05$). The *P* value indicates the significance of differences in the mean values (versus the indicated group). Source data are provided as a Source Data file.

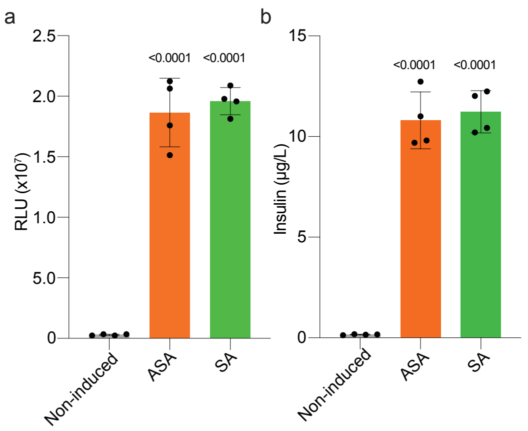


**Supplementary Figure 15 | NLuc and insulin production by alginate-encapsulated HEK-ASPIRIN cells. a,b,** The microencapsulated cells were cultured in 24-well plates with DMEM medium supplemented with 10% fetal bovine serum and stimulated with ASA or SA at the concentration of 200 µM. Subsequently, the levels of NLuc (**a**) and insulin (**b**) in the culture supernatant were quantified following a 24-hour induction period. Each column represents the mean ± standard deviation of four determinations, as indicated by dots. Statistical significance was analysed by means of a two-sided unpaired t test. The *P* value reflects the significance of differences in the mean values compared to the non-induced group. Source data are provided as a Source Data file.

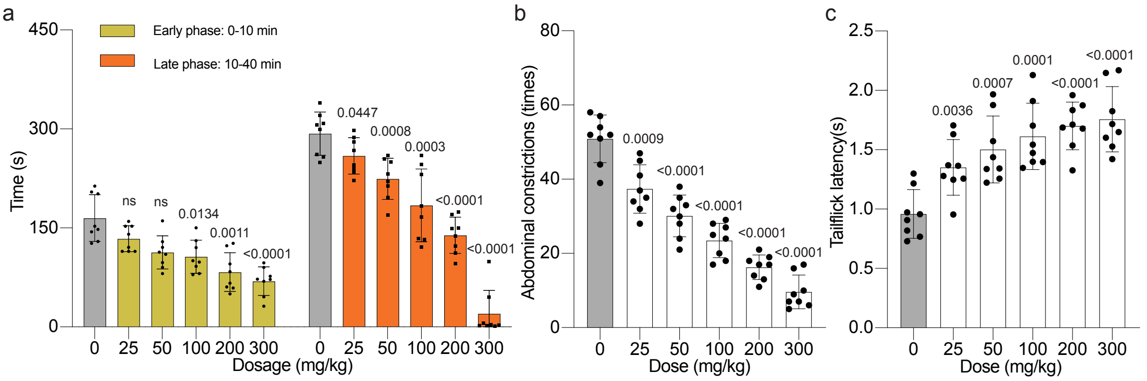


**Supplementary Figure 16. Evaluation of anti-nociceptive activity of ASA *in vivo*. a**, Anti-nociceptive effect of ASA on formalin-induced hindpaw licking behavior in WT mice. Mouse behavior was monitored during the "early phase" (0-10 min after intraplantar formalin injection) and "late phase" (10-40 min after injection). Each mouse received 10 µl of 5% formalin in the right hindpaw 15 min after oral administration of ASA at the indicated concentrations. Licking time was recorded over 40 min after formalin injection. **b**, Anti-nociceptive effect of ASA on abdominal constrictions induced by acetic acid in WT mice. ASA was orally administered at the indicated concentration. 15 min later, freshly prepared acetic acid (2.5% in saline, 200 µl/mice) was intraperitoneally injected into the mice. Abdominal constrictions of the mice were counted over a 30-min period. **c**, Anti-nociceptive effect of ASA on tail withdrawal latency to a noxious thermal stimulus in WT mice. Tail withdrawal latency behavior was monitored 15 min post oral administration of ASA at the indicated concentrations. All data are presented as means ± SD; n = 8. Statistical significance was analysed by means of a two-sided unpaired t test. The *P* values denote the significance of differences in mean values versus the non-induced control or indicated groups. "ns" indicates not significant (*P* value > 0.05) compared to non-induced control or indicated groups. Grey columns represent the untreated control groups. Source data are provided as a Source Data file.

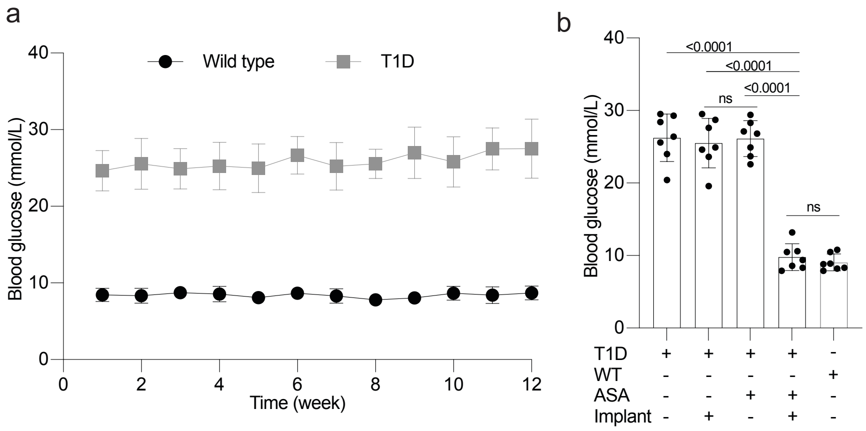


**Supplementary Figure 17 | Blood glucose monitoring in T1D and wild-type mice before experiments.** Monitoring of glycemia over four consecutive weeks in T1D and wild-type mice. Monitoring of fasting glycemia after five days treatment with or without ASA in the serum of WT without cell implants, T1D negative control with or without cell implants, stimulated (ASA (+))T1D mice with or without cell implants. Statistical significance was analysed by means of an unpaired t test. The experiment was performed one time. Data points represent mean ± SD; n = 7. The *P* values denote the significance of differences in mean values versus the non-induced control or indicated groups. Statistical significance was analysed by means of two-sided two-way ANOVA with Tukey's test. "ns" indicates not significant (*P* value > 0.05) compared to the non-induced control or indicated groups. Source data are provided as a Source Data file.

**Supplementary Table 1.** Chemicals used in this study.

| **Names** | **Linear Formula** | **Cat. No.** | **Company** |
|---|---|---|---|
| Acetylsalicylic acid (aspirin; ASA) | 2-$(CH_3CO_2)C_6H_4CO_2H$ | A2093-100G | MERCK |
| Salicylic acid (SA) | 2-$(HO)C_6H_4CO_2H$ | 247588-100G | MERCK |
| Benzoic acid (BA) | $C_6H_5COOH$ | 8222570100 | MERCK |
| 3-Hydroxybenzoic acid (3HBA) | $HOC_6H_4CO_2H$ | H20008-5G | MERCK |
| 4-Hydroxybenzoic acid (4HBA) | $HOC_6H_4CO_2H$ | 8218140005 | MERCK |
| o-Toluic acid (OTA) | $CH_3C_6H_4CO_2H$ | T36404-100G | MERCK |
| 4-Hydroxy-1,3-benzenedicarboxylic acid (Salicylic Acid Related Compound B, 4HBDA) | $HOC_6H_3$-1,3-$(CO_2H)_2$ | 1609024-100MG | MERCK |
| Phthalic acid (PA) | $C_6H_4$-1,2-$(CO_2H)_2$ | 8222980100 | MERCK |
| 2,3-Dihydroxybenzoic acid | $(HO)_2C_6H_3CO_2H$ | 41398-100MG | MERCK |
| 2,5-Dihydroxybenzoic acid | $(HO)_2C_6H_3CO_2H$ | 8417450050 | MERCK |
| 3,4-Dihydroxybenzoic Acid (Protocatechuic acid, PCA) | $(HO)_2C_6H_3CO_2H$ | W443095 | MERCK |
| Salicylic acid acyl-b-D-glucuronide (SAADG) | $C_{13}H_{14}O_9$ | TRC-S088135-10MG | LGC Group |

| | | | |
|---|---|---|---|
| Salicylic acid b-D-O-glucuronide (SADOG) | $C_{13}H_{14}O_9$ | TRC-S088130-5MG | LGC Group |
| p-Toluic acid | $CH_3C_6H_4CO_2H$ | T36803-5G | MERCK |
| Methyl salicylate (MSC) | 2-$(HO)C_6H_4CO_2CH_3$ | M6752-250ML | MERCK |
| 4-Hydroxy-1,3-benzenedicarboxylic acid | $HOC_6H_3$-1,3-$(CO_2H)_2$ | 797057-250MG | MERCK |
| 4-Hydroxy-2-methylbenzoic acid (HMBA) | $HOC_6H3(CH_3)CO_2H$ | 653160-10G | MERCK |
| 5-Hydroxy-2-methylbenzoic acid (5H2MBA) | $C_8H_8O_3$ | 696366 | MERCK |
| Disalicyl succinate (DSS) | $C_{18}H_{14}O_8$ | R288217-250MG | MERCK |
| 1,4-Dihydro-2-methylbenzoic acid (DHMBA) | $C_8H_{10}O_2$ | 300357-1G | MERCK |
| 2,5-Dihydroxybenzoic acid (DDBA) | $(HO)_2C_6H_3CO_2H$ | 8417450050 | MERCK |
| m-Toluic acid (MTA) or 3-methylbenzoic acid (3MBA) | $CH_3C_6H_4CO_2H$ | T36609-5G | MERCK |
| Gentisuric Acid | $C_9H_9NO_5$ | ABA35124 | BIOSYNTH |
| o-Hydroxyhippuric acid (Salicyluric acid, SCA) | $C_9H_9NO_4$ | 49861 | MERCK |
| 1,2,3-Benzothiadiazole | $C_8H_6N_2OS_2$ | 32820-100MG | MERCK |

| | | | |
|---|---|---|---|
| 2,6-Dichloroisonicotinic acid | $C_6H_3Cl_2NO_2$ | 456543-1G | MERCK |

**References:**


1 Angiolillo, D. J. *et al.* Pharmacokinetic and pharmacodynamic profile of a novel phospholipid aspirin formulation. *Clinical Pharmacokinetics* **61**, 465-479 (2022).

2 Ballerini, P. *et al.* Inflammation and cancer: from the development of personalized indicators to novel therapeutic strategies. *Frontiers in Pharmacology* **13**, 838079 (2022).

3 Rainsford, K. D. *Aspirin and the Salicylates*. (Elsevier, 2013).

4 Bojić, M., Sedgeman, C. A., Nagy, L. D. & Guengerich, F. P. Aromatic hydroxylation of salicylic acid and aspirin by human cytochromes P450. *European journal of pharmaceutical sciences* **73**, 49-56 (2015).

5 Uzzaman, M., Chowdhury, M. K. & Belal Hossen, M. Thermochemical, molecular docking and ADMET studies of aspirin metabolites. *Front. Drug, Chem. Clin. Res* **2**, 1-5 (2019).

Source data file for **Supplementary Figure 8** (Uncropped scans of all blots)

Uncropped scan of blots for **Anti-HA-input**:

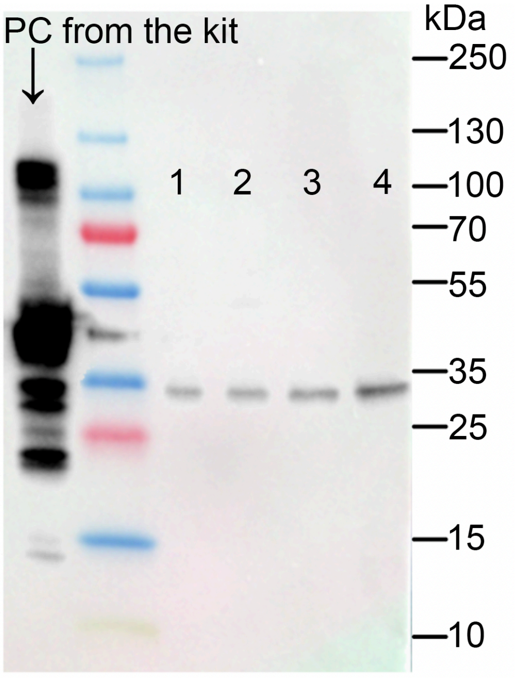


Uncropped scan of blots for **Anti-FLAG-input**:

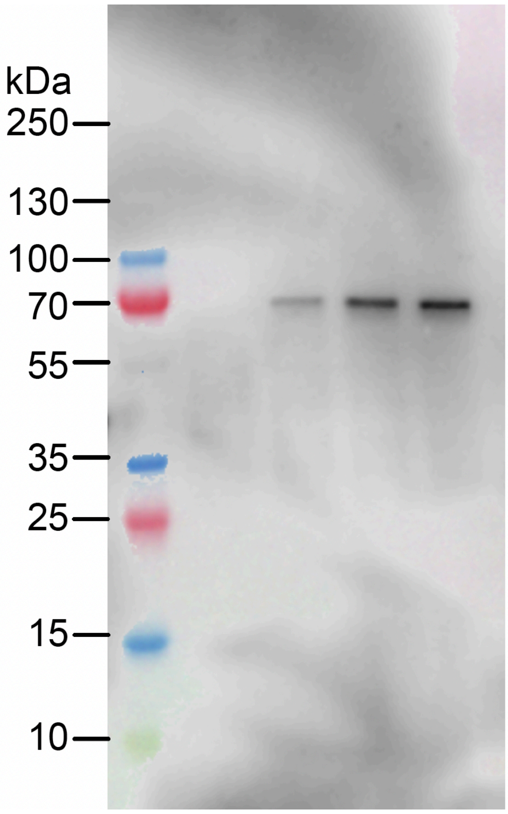

Uncropped scan of blots for **Anti-HA-IP**:

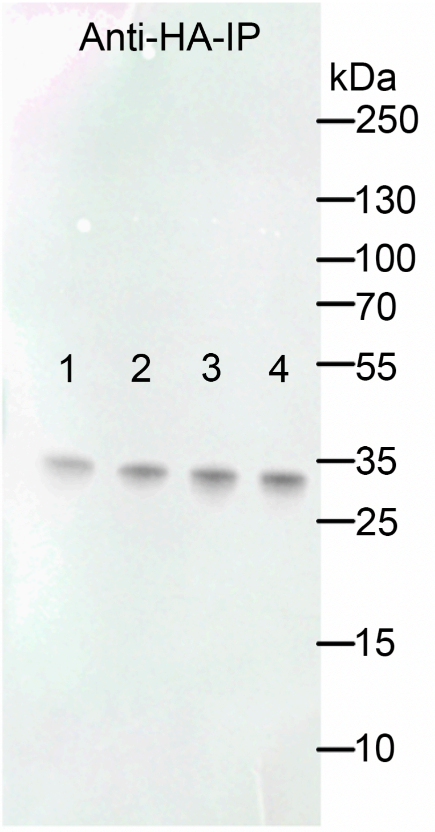


Uncropped scan of blots for **Anti-FLAG-IP**:

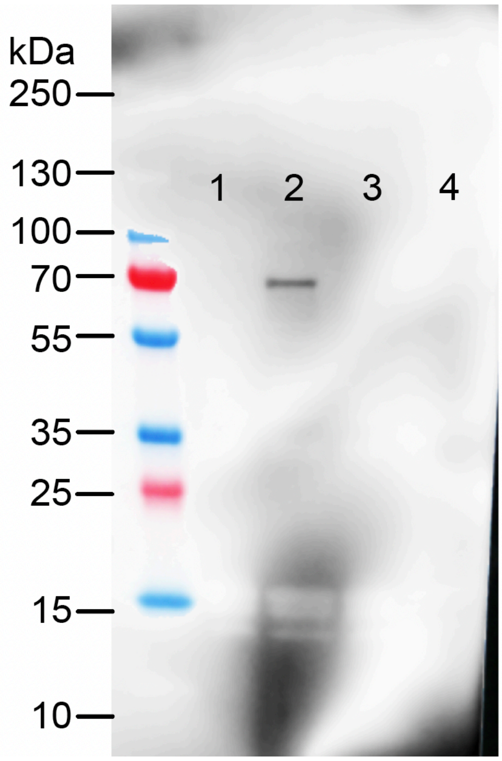

Supplement: Supplementary file 1 — Supplementary Information [file 41467_2025_57275_MOESM1_ESM.pdf]
