# Supplementary material for: Aspirin-responsive gene switch regulating therapeutic protein expression
Source: Nat Commun. 2025 Feb 27;16:2028. doi: 10.1038/s41467-025-57275-x (PMC11868571; doi:10.1038/s41467-025-57275-x)
Supplement: Supplementary file 2 — Description of Additional Supplementary Information [file 41467_2025_57275_MOESM2_ESM.docx]

**Description of Additional Supplementary Files**

File Name: Supplementary Data 1

Description: Plasmids used and designed in this study.

File Name: Supplementary Data 2

Description: Oligonucleotide sequences used in this work.

File Name: Supplementary Data 3

Description: Primers used for qPCR analysis.

File Name: Supplementary Data 4

Description: Full-length sequences of the key plasmids in this study.
