# Supplementary material for: Aspirin-responsive gene switch regulating therapeutic protein expression
Source: Nat Commun. 2025 Feb 27;16:2028. doi: 10.1038/s41467-025-57275-x (PMC11868571; doi:10.1038/s41467-025-57275-x)
Supplement: Supplementary file 7 — Reporting Summary [file 41467_2025_57275_MOESM7_ESM.pdf]

nature portfolio

Corresponding author(s): Martin Fussenegger

Last updated by author(s): Feb 6, 2025

Reporting Summary

Nature Portfolio wishes to improve the reproducibility of the work that we publish. This form provides structure for consistency and transparency in reporting. For further information on Nature Portfolio policies, see our Editorial Policies and the Editorial Policy Checklist.

Statistics

For all statistical analyses, confirm that the following items are present in the figure legend, table legend, main text, or Methods section.

n/a	Confirmed	
☐	☒	The exact sample size (*n*) for each experimental group/condition, given as a discrete number and unit of measurement
☐	☒	A statement on whether measurements were taken from distinct samples or whether the same sample was measured repeatedly
☐	☒	The statistical test(s) used AND whether they are one- or two-sided<br>*Only common tests should be described solely by name; describe more complex techniques in the Methods section.*
☐	☒	A description of all covariates tested
☐	☒	A description of any assumptions or corrections, such as tests of normality and adjustment for multiple comparisons
☐	☒	A full description of the statistical parameters including central tendency (e.g. means) or other basic estimates (e.g. regression coefficient) AND variation (e.g. standard deviation) or associated estimates of uncertainty (e.g. confidence intervals)
☐	☒	For null hypothesis testing, the test statistic (e.g. *F*, *t*, *r*) with confidence intervals, effect sizes, degrees of freedom and *P* value noted<br>*Give P values as exact values whenever suitable.*
☒	☐	For Bayesian analysis, information on the choice of priors and Markov chain Monte Carlo settings
☒	☐	For hierarchical and complex designs, identification of the appropriate level for tests and full reporting of outcomes
☒	☐	Estimates of effect sizes (e.g. Cohen's *d*, Pearson's *r*), indicating how they were calculated

Our web collection on statistics for biologists contains articles on many of the points above.

Software and code

Policy information about availability of computer code

Data collection	All the absorbance and luminescence data were collected using Tecan SPARK plate reader (Tecan Group AG, Switzerland). Flow cytometry analysis was performed and analyzed on a FACSAria Fusion Cell Sorter, Becton Dickinson, New Jersey, USA. microscopic imaging was performed using a Leica SP8 confocal microscope equipped with a laser system. The RNA samples were quantified using a NanoDrop 2000 spectrophotometer (Thermo Fisher). Western signals were visualized using the Pierce ECL Western Blotting Substrate (ThermoFisher, cat. no. 32106) and a chemiluminescence detection system (Azure 400, Azure Biosystems, USA). Quantitative PCR was performed using the QuantStudio 3 system (Thermo Fisher). Blood glucose levels in mice were measured using clinically licensed Contour®Next test strips and the Contour®Next ONE reader (Ascensia Diabetes Care, Switzerland).
Data analysis	For statistical evaluations involving multiple comparisons, GraphPad Prism 8 (v 9.2.0, GraphPad Software Inc.) and Microsoft Excel (v16.51, Microsoft) were employed. A two-tailed, unpaired Student's t-test and one-way or two-way analysis of variance (ANOVA) were utilized to determine the statistical significance of differences. All the figures were produced using Illustrator (V28.2, Adobe, USA). Chemical structures were drawn using ChemDraw (v20.0.0.38, PerkinElmer).

For manuscripts utilizing custom algorithms or software that are central to the research but not yet described in published literature, software must be made available to editors and reviewers. We strongly encourage code deposition in a community repository (e.g. GitHub). See the Nature Portfolio guidelines for submitting code & software for further information.

Data

Policy information about availability of data

All manuscripts must include a data availability statement. This statement should provide the following information, where applicable:

- Accession codes, unique identifiers, or web links for publicly available datasets
- A description of any restrictions on data availability
- For clinical datasets or third party data, please ensure that the statement adheres to our policy

The authors declare that all the data supporting the findings of this study are available within the paper, supplementary materials and Source Data file. All original plasmids listed in Supplementary Data 1 are available if it is requested for scientific purposes. Any additional inquiries should be addressed to Jinbo Huang or Martin Fussenegger (corresponding author). The full-length sequences of the key plasmids: pJH138, pJH140, pJH142, pJH144, pJH196, pJH230, pJH231, pJH2012, and pJH2036, are available in Supplementary Data 4. Source data are provided with this paper.

Research involving human participants, their data, or biological material

Policy information about studies with human participants or human data. See also policy information about sex, gender (identity/presentation), and sexual orientation and race, ethnicity and racism.

Reporting on sex and gender	The authors declare that no human research participants were involved in this study.
Reporting on race, ethnicity, or other socially relevant groupings	The authors declare that no human research participants were involved in this study.
Population characteristics	The authors declare that no human research participants were involved in this study.
Recruitment	The authors declare that no human research participants were involved in this study.
Ethics oversight	The authors declare that no human research participants were involved in this study.

Note that full information on the approval of the study protocol must also be provided in the manuscript.

Field-specific reporting

Please select the one below that is the best fit for your research. If you are not sure, read the appropriate sections before making your selection.

☒ Life sciences ☐ Behavioural & social sciences ☐ Ecological, evolutionary & environmental sciences

For a reference copy of the document with all sections, see nature.com/documents/nr-reporting-summary-flat.pdf

Life sciences study design

All studies must disclose on these points even when the disclosure is negative.

Sample size	In practice, the sample size used in this study is usually determined based on the need for it to offer sufficient statistical power, and the time, cost, or convenience of collecting the data. No specific statistical methods were used to predetermine sample size.
Data exclusions	No data were excluded from the analyses.
Replication	All experiments in this study were successfully reproduced at least twice.
Randomization	The experiments were not randomized.
Blinding	The investigators were not blinded to allocation during experiments and outcome assessment.

Reporting for specific materials, systems and methods

We require information from authors about some types of materials, experimental systems and methods used in many studies. Here, indicate whether each material, system or method listed is relevant to your study. If you are not sure if a list item applies to your research, read the appropriate section before selecting a response.

Materials & experimental systems

n/a	Involved in the study
☐	☒ Antibodies
☐	☒ Eukaryotic cell lines
☒	☐ Palaeontology and archaeology
☐	☒ Animals and other organisms
☒	☐ Clinical data
☒	☐ Dual use research of concern
☒	☐ Plants

Methods

n/a	Involved in the study
☒	☐ ChIP-seq
☐	☒ Flow cytometry
☒	☐ MRI-based neuroimaging

Antibodies

Antibodies used	pJH275, Anti-FLAG, Abcam, ab205606; Western blot (1:1000), lot.no. 1043065-26;<br>pJH275, Anti-FLAG, Sigma-Aldrich, F1804-50UG; Western Blot (1:2000),SLCN3722;<br>pJH272, Anti-HA antibody: Abcam, ab236632; Western blot (1:1000), lot.no. 1006247-28;<br>Goat Anti-mouse IgG: Abcam, AB205719, Western blot (1:2000); lot.no. 1093055-3;<br>Goat Anti-rabbit IgG: Sigma-Aldrich, A6154-1ML, Western blot (1:5000); lot.no. 1003358441;<br>Anti-GAPDH, Abcam, AB9485, Western blot (1:2000), lot.no. 1064471-1.
Validation	All the commercially available antibodies used in this study were validated by the manufacturers and/or previous publications through Western blot.<br>Anti-FLAG, Abcam, ab205606 (https://www.abcam.com/en-us/products/primary-antibodies/ddddk-tag-binds-to-flag-tag-sequence-antibody-epr20018-251-ab205606) ;<br>Anti-FLAG, Sigma-Aldrich, F1804-50UG (https://www.sigmaaldrich.com/CH/en/product/sigma/f1804#product-documentation);<br>Anti-HA antibody: Abcam, ab236632 (https://www.abcam.com/en-us/products/primary-antibodies/ha-tag-antibody-epr22819-101-ab236632);<br>Goat Anti-mouse IgG: Abcam, AB205719 (https://www.abcam.com/en-us/products/secondary-antibodies/goat-mouse-igg-h-l-hrp-ab205719);<br>Goat Anti-rabbit IgG: Sigma-Aldrich, A6154-1ML (https://www.sigmaaldrich.com/CH/en/product/sigma/a6154);<br>Anti-GAPDH, Abcam, AB9485 (https://www.abcam.com/en-us/products/primary-antibodies/gapdh-antibody-loading-control-ab9485).

Eukaryotic cell lines

Policy information about cell lines and Sex and Gender in Research

Cell line source(s)	Cell lines used in this study: Human embryonic kidney cells (HEK-293T, ATCC: CRL-11268), baby hamster kidney cells (BHK-21, ATCC: CCL-10), Chinese hamster ovary cells (CHO-K1, ATCC: CCL-61), human telomerase-immortalized mesenchymal stem cells (hMSC-TERT, Simonsen et al., Nature Biotechnology, 2002), human cervical adenocarcinoma cells (HeLa, ATCC: CCL-2), human colorectal adenocarcinoma cells (Caco-2, ATCC: HTB-37), and human liver cancer cells (Hep G2, ATCC: CRL-11997).
Authentication	All the cell lines used in this study were authenticated by the supplier and the authorities of the Department of Biosystems Science and Engineering (D-BSSE) of the ETH Zurich in Basel, Switzerland.
Mycoplasma contamination	The authors declare that all the cell lines in this study were tested negative for mycoplasma contamination.
Commonly misidentified lines<br>(See ICLAC register)	No commonly misidentified cell lines were used in this study.

Animals and other research organisms

Policy information about studies involving animals; ARRIVE guidelines recommended for reporting animal research, and Sex and Gender in Research

Laboratory animals	The 8-week-old wild-type male Swiss mice (C57BL/6J, Janvier Labs) were used in this study.
Wild animals	The authors declare that no wild animals were used in this study.
Reporting on sex	The male mice used in this study were randomly selected by following previous studies (Bai et al., Nature Medicine, 2019; Krawczyk et al., Science, 2020; Zhou et al., Nature Biotechnology, 2021; Chen et al., Nature Chemical Biology; Schneider et al., Science Advances, 2021, Huang et al., Nature metabolism, 2023), as well as due to the convenience of husbandry with the same sex. Sex was not considered in study design. No data disaggregated for sex were collected.
Field-collected samples	The authors declare that no field-collected samples were used in this study.
Ethics oversight	This study was conducted in accordance with all relevant ethical regulations. For animal studies, all animal experiments fully adhered to French or Chinese animal welfare legislation. The experiments were approved by the French Republic (project no. DR2018-40v5

and APAFIS no. 16753), and conducted by Jinbo Huang, Shuai Xue, and Ghislaine Charpin-El Hamri (no. 69266309) at the University of Lyon, Institut Universitaire de Technologie (IUT, F69622 Villeurbanne, France), or performed by Ting Gao and Shuai Xue according to the protocol (Protocol ID: AP#24-088-XMQ) approved by the Institutional Animal Care and Use Committee (IACUC) of Westlake University and in accordance with the Animal Care Guidelines of the Ministry of Science and Technology of the People's Republic of China. In this study, to keep standard metabolic activity, and avoid the effect of heredity, breed, age, sex or weight, we used only male mice to minimize variability associated with hormonal fluctuations in females, ensuring consistency in our results. This approach aligns with previous studies in the field and allows for a more controlled investigation of diabetes.

Note that full information on the approval of the study protocol must also be provided in the manuscript.

Plants

Seed stocks	The authors declare that no plant-related research in this study.
Novel plant genotypes	The authors declare that no plant-related research in this study.
Authentication	The authors declare that no plant-related research in this study.

Flow Cytometry

Plots

Confirm that:

- [x] The axis labels state the marker and fluorochrome used (e.g. CD4-FITC).
- [x] The axis scales are clearly visible. Include numbers along axes only for bottom left plot of group (a 'group' is an analysis of identical markers).
- [x] All plots are contour plots with outliers or pseudocolor plots.
- [x] A numerical value for number of cells or percentage (with statistics) is provided.

Methodology

Sample preparation	The sample preparation is described in details in the Methods section of the manuscript. Briefly, 3.5x105 HEK-293T cells were cultured in 6-well plates for 24 h. To establish the stable cell line, cells were co-transfected with 1200 ng of pJH230, 300 ng of pJH231, 1200 ng of pJH2036, and 300 ng of pJH42 (PhCMV-SB100X-pA), which encodes constitutive expression of the Sleeping Beauty transposase SB100X. At 24 hours post-transfection, cells were transferred to fresh medium containing puromycin (1 µg/ml), blasticidin (10 µg/ml) and zeocin (50 µg/ml) for a three-day antibiotic selection process. Subsequently, the polyclonal population of genetically engineered cells was suspended in DMEM medium and sorted in a fluorescence-activated cell sorting (FACS) system (BD Biosciences) based on the yPET (517/530), mRuby (558/605), and iRFP (690/713) fluorescence signals. The triple-positive cell population was isolated as single cells in 96-well plates and maintained in triple antibiotic-containing selection medium. Following two weeks of clonal expansion, monoclonal cell lines were screened by the addition of ASA, and the most promising cell line was selected for subsequent experiments. The selection was further validated through fluorescence-based flow cytometry, which confirmed the presence of triple-positive fluorescence signals in the chosen optimal cell line.
Instrument	Flow cytometry analysis was performed on a FACSAria Fusion Cell Sorter, Becton Dickinson, New Jersey, USA
Software	Flow cytometry data were analyzed using FACSAria Fusion Cell Sorter, Becton Dickinson, New Jersey, USA
Cell population abundance	Using fluorescent output, the triple-positive (yPET (517/530), mRuby (558/605), and iRFP (690/713) fluorescence signals) cell population was isolated as single cells in 96-well plates and maintained in triple antibiotic-containing selection medium. The selected monoclonal cell line was further validated through fluorescence-based flow cytometry, which confirmed the presence of triple-positive fluorescence signals.
Gating strategy	A comprehensive report is attached in supplementary file. Gating for positive cells was performed based on HEK-293T cells expressing no fluorophore.

- [x] Tick this box to confirm that a figure exemplifying the gating strategy is provided in the Supplementary Information.
